# Prognostic Impact of Cirrhosis in Patients with Intrahepatic Cholangiocarcinoma following Hepatic Resection

**DOI:** 10.1155/2017/6543423

**Published:** 2017-11-12

**Authors:** Seogsong Jeong, Lei Gao, Ying Tong, Lei Xia, Ning Xu, Meng Sha, Jianjun Zhang, Xiaoni Kong, Jinyang Gu, Qiang Xia

**Affiliations:** ^1^Department of Liver Surgery, Renji Hospital, School of Medicine, Shanghai Jiao Tong University, Shanghai 200127, China; ^2^Institute of Liver Diseases, 81st Hospital of People's Liberation Army, Nanjing 210002, China

## Abstract

**Background:**

Prognostic impact of cirrhosis in patients with intrahepatic cholangiocarcinoma (ICC) upon hepatic resection remains unclear due to lack of studies in the literature.

**Methods:**

A total of 106 resected patients with ICC were reviewed, including 25 patients (23.6%) with cirrhosis and 81 noncirrhotic patients (76.4%). Subgroups of cirrhotic patients with and without hepatitis B virus (HBV) infection were studied.

**Results:**

The impact of cirrhosis on the overall survival (OS) (hazard ratio [HR], 0.901; 95% confidence interval [CI], 0.510 to 1.592; *P* = 0.720) and the relapse-free survival (RFS) (HR, 0.889; 95% CI, 0.509 to 1.552; *P* = 0.678) revealed no statistical significance. Furthermore, HBV-associated cirrhotic patients and the other cirrhotic patients demonstrated no statistical difference on survival outcomes (1 yr OS, 60.0% versus 70.0%; 5 yr OS, 10.0% versus 0%; *P* = 0.744; 1 yr RFS, 53.3% versus 30.0%; 5 yr RFS, 10.0% versus 0%; *P* = 0.279). In patients with cirrhosis, tumor size larger than 5 cm was found to be the foremost factor that was independently associated with poor prognosis.

**Conclusion:**

The presence of liver cirrhosis did not significantly affect prognosis of patients with ICC after resection. Downstaging modality may be in need for patients with ICC underlying cirrhosis, which remains to be validated in future studies.

## 1. Introduction

Intrahepatic cholangiocarcinoma (ICC) is the second most common primary liver cancer after hepatocellular carcinoma (HCC) that originates from epithelial cells of intrahepatic bile ducts [1]. To date, surgical resection remains the only curative treatment that has been generally recognized for ICC [2]. However, clinical manifestation of ICC is usually late and unspecific, leading most ICC to be advanced at the time of diagnosis [3]. Consequently, approximately one-third of patients with ICC are not recommended to be candidates for hepatic resection due to high postoperative recurrence rates, even after extended hepatectomy [4]. Another major concern that limits patients with ICC to receive hepatic resection is underlying liver disease, including primary biliary cirrhosis in western countries and hepatitis B virus- (HBV-) associated cirrhosis in eastern countries [5]. A few previous publications indicated that the presence of cirrhosis was independently associated with reduced long-term survival outcomes in patients with HCC [6, 7]. In addition, Li et al. [8] demonstrated that cirrhosis is an independent predictor for poor prognosis in patients with ICC after resection. In another study of 514 patients with ICC who underwent hepatic resection, cirrhosis was also found to be an unfavorable prognostic factor so that they subjected the presence of cirrhosis into the nomogram predicting prognosis [9]. On the contrary, Zhang et al. [10] reported that the presence of cirrhosis showed no impact in the prognosis of patients with ICC (*P* = 0.730). Therefore, the exact relationship of liver cirrhosis with the prognosis of ICC remains to be further elucidated.

Herein, we conducted the present study to evaluate prognostic impact of liver cirrhosis on ICC. All the enrolled patients were divided according to the presence of liver cirrhosis as well as HBV infection, which would especially focus on HBV-associated liver cirrhosis frequently in China. Furthermore, a statistical analysis was carried out to identify foremost factors significantly affecting long-term survival outcomes of cirrhotic patients.

## 2. Methods

### 2.1. Patients

From January 2007 to July 2015, 106 consecutive patients with ICC underwent hepatic resection at the Department of Liver Surgery, Renji Hospital (Shanghai, China). The patients were included according to the following criteria: (1) pathological confirmation of ICC; (2) single type of tumor; (3) Child-Pugh score of A or B; (4) hepatic resection, and (5) not having distant metastasis at the time of surgery.

### 2.2. Definition of Subgroups

The enrolled patients were stratified into 4 subgroups according to the presence of HBV infection and liver cirrhosis. The first group (Group 1; *n* = 15) was comprised of cirrhotic patients with HBV infection, and noncirrhotic patients with HBV-associated ICC were defined as Group 2 (*n* = 27). Group 3 (*n* = 10) was consisted of the cirrhotic patients without seropositivity of HBV infection, and the others were subjected into Group 4 (*n* = 54).

### 2.3. Data Collection

Prospectively collected ICC database was used for the description of baseline characteristics and survival analyses. HBV infection was defined as positivity of either hepatitis B surface antigen (HBsAg) or hepatitis B core antibody (HBcAb) and confirmed by serological examinations. Liver cirrhosis was collected from results of imaging studies and further confirmed by Sirius Red staining from liver specimens after hepatic resection. Preoperative levels of alpha fetoprotein (AFP) and carbohydrate antigen (CA) 19-9 were collected within one week before operation. Tumor size was defined as a maximal diameter of the principal tumor nodule. Histologic grade was classified by the Edmondson-Steiner criteria ((I) well; (II) moderate; (III) poor) [11]. The follow-up investigation was carried out ranging from 17 to 118 months. The endpoints of the study were the survival outcomes of the patients.

### 2.4. Surgical Procedures

All the patients underwent hepatic resection with regional lymph node dissection. The types of resection included minor hepatectomy (partial or less than a half of liver) and major hepatectomy (hemihepatectomy or extended hemihepatectomy). The indication for major hepatectomy was an indocyanine green clearance rate at 15 min rate of less than 10%. All the surgical operations were performed with informed consents.

### 2.5. Statistical Analysis

SPSS for Windows version 19.0 (SPSS Inc., Chicago, Illinois, US) was applied in statistical analyses. Categorical data were analyzed with the use of Pearson's chi-square test. Survival curves were presented with the use of Kaplan-Meier method. Between-group differences were evaluated by Log-rank method. The univariate and multivariate analyses were applied in assessing prognostic value of variables and identifying independent prognostic factors, which were presented with hazard ratio (HR) and 95% confidence interval (CI). Statistical significance was defined as a *P* value of less than 0.05.

## 3. Results

### 3.1. Patient Characteristics

The mean age was 60.0 ± 10.34 with a 58.5% of male distribution (Table 1). The age was significantly younger in HBV-associated cirrhotic patients compared with the cirrhotic patients without HBV infection (54.9 ± 7.4 yrs versus 71.2 ± 8.4 yrs, *P* < 0.001) with a predominance of male patients (*P* = 0.013; Table 2). The mean age of cirrhotic patients without HBV infection revealed to be older than the noncirrhotic patients without HBV infection (71.2 ± 8.4 yrs versus 62.0 ± 9.8 yrs, *P* = 0.011). Elevation of AFP was found in 29 patients (26.4%), whereas more than two-thirds (68.9%) of the patients had an elevated level of CA19-9. Minor and major hepatectomy were performed in 49 (45.3%) and 58 (54.7%) patients, respectively. More than a half of ICC patients (57.5%) had a larger than 5 cm of maximal diameter of tumor. Eighty-nine patients (84.0%) showed a single tumor nodule and the other 17 patients (16.0%) presented with multiple nodules. Almost all the patients (*n* = 100, 94.3%) displayed Child-Pugh score of A. Fifty-two (49.1%) patients belonged to well or moderate differentiation, whereas the other 54 (50.9%) were poorly differentiated. Vascular invasion and lymph node metastasis were found in 28 (26.4%) and 48 (45.3%) patients, respectively. Further specific between-group differences are shown in Table 2.

### 3.2. Prognostic Factors of All Resected Patients with ICC

As for the cumulative OS of all the resected patients, HBsAg, preoperative level of CA19-9, tumor size, Child-Pugh score, vascular invasion, and lymph node metastasis revealed significance in the univariate analysis and were employed into the multivariate analysis. Tumor size larger than 5 cm (HR, 1.923; 95% CI, 1.147 to 3.226; *P* = 0.013), Child-Pugh score of B (HR, 3.349; 95% CI, 1.387 to 8.090; *P* = 0.007), vascular invasion (HR, 1.867; 95% CI, 1.103 to 3.159; *P* = 0.020), and lymph node metastasis (HR, 2.790; 95% CI: 1.628 to 4.781; *P* < 0.001) were found to be independently associated with decreased OS (Table 3).

Seropositivity of HBsAg, tumor size, Child-Pugh score, lymph node metastasis, and tumor number were found to affect RFS. Among them, the seropositivity of HBsAg was associated with prolonged RFS (HR, 0.505; 95% CI, 0.279 to 0.914; *P* = 0.024), whereas tumor size larger than 5 cm (HR, 1.947; 95% CI, 1.177 to 3.219; *P* = 0.009), Child-Pugh score of B (HR, 3.067; 95% CI, 1.293 to 7.275; *P* = 0.011), and lymph node metastasis (HR, 2.188; 95% CI, 1.310 to 3.654; *P* = 0.003) negatively influenced the RFS.

### 3.3. Survival Outcomes

**Figure 1:**
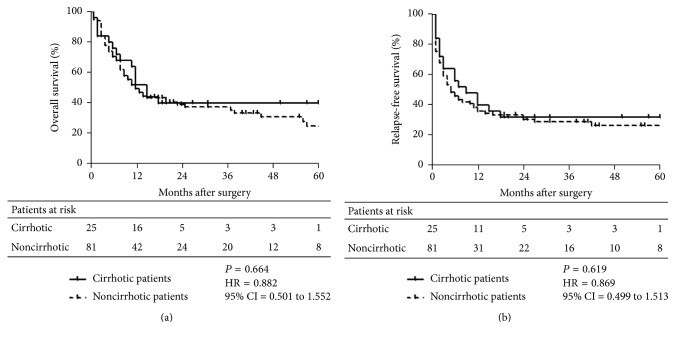
Survival outcomes of the patients with and without cirrhosis. Shown are the impact of cirrhosis on the OS (Panel (a)) and RFS (Panel (b)) of the entire cohort. The data revealed that the presence of cirrhosis showed no association with the OS (HR, 0.882; 95% CI, 0.501 to 1.552; *P* = 0.664) and RFS (HR, 0.869; 95% CI, 0.499 to 1.513; *P* = 0.619) of the patients.

**Figure 2:**
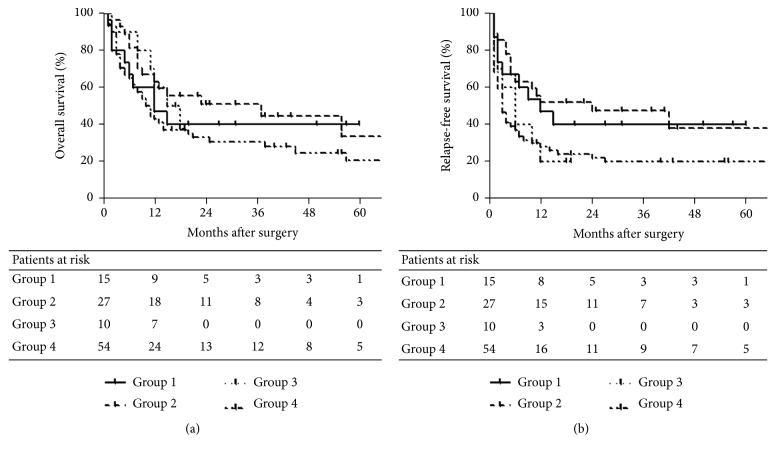
Survival curves of the patients according to the presence of HBV infection and cirrhosis. Shown are the survival curves of the HBV-associated cirrhotic patients (Group 1), HBV-associated noncirrhotic patients (Group 2), HBV-negative cirrhotic patients (Group 3), and HBV-negative noncirrhotic patients (Group 4) on the OS (Panel (a)) and RFS (Panel (b)). The curves demonstrated no significant between-group differences except for Group 2 and Group 4 in the RFS (*P* = 0.011). The most favorable prognosis was found in Group 2 (1-yr OS, 66.7%; 5-yr OS, 16.7%; median survival for OS, 37 months; 1-yr RFS, 55.6%; 5-yr RFS, 16.7%; median survival for RFS, 24 months) and Group 4 showed the worst prognosis (1-yr OS, 44.4%; 5-yr OS, 11.1%; median survival for OS, 10.5 months; 1-yr RFS, 29.6%; 5-yr RFS, 10.4%; median survival for RFS, 3 months).

Survival outcomes of the cirrhotic patients and noncirrhotic patients are presented in Figure 1. As shown in Figures 1(a) and 1(b), the presence of cirrhosis revealed no significant impact on the OS (HR, 0.882; 95% CI, 0.501 to 1.552; *P* = 0.664) and RFS (HR, 0.869; 95% CI, 0.499 to 1.513; *P* = 0.619). When the patients were stratified into 4 groups, the OS indicated no significant between-group differences, whereas the RFS showed a statistically significant difference between Groups 2 and 4 (1-yr, 55.6% versus 29.6%; 5-yr, 16.7% versus 10.4; *P* = 0.011; Table 4; Figure 2).

### 3.4. Prognostic Factors of Cirrhotic Patients with ICC

Minor hepatectomy (versus major hepatectomy), tumor size > 5 cm (versus ≤5 cm), and lymph node metastasis showed a significant association with the survival outcomes in the univariate analysis. In addition, major hepatectomy showed a HR of 4.194, 95% CI of 1.447 to 12.093, and a *P* value of 0.008 for the OS, and a HR of 3.159, 95% CI of 1.164 to 8.576, and a *P* value of 0.024 for the RFS. However, the type of hepatectomy and lymph node metastasis revealed no significantly independent association with both the OS and RFS. Only the tumor size larger than 5 cm was found to be independently reduced in the OS (HR, 16.435; 95% CI, 2.211 to 122.179; *P* = 0.006) and RFS (HR, 10.264; 95% CI, 1.881 to 55.987; *P* = 0.007) of the cirrhotic patients (Table 5). Furthermore, among the 11 cirrhotic patients with tumor size larger than 5 cm, 8 patients had relapse of the tumor within 6 months after surgical operation and the other 3 patients developed recurrence at the 9th, 10th, and 12th month.

## 4. Discussion

Although hepatic resection provides patients with ICC an opportunity of long-term survival, the prognosis of this group of patients remains dismal [12, 13]. In eastern countries, numerous patients present with underlying liver disease, such as liver cirrhosis, due to a high prevalence of viral hepatitis that limits surgical approaches [14]. As known widely, liver cirrhosis may contribute to severe complications, including deterioration of liver function and gastrointestinal bleeding [15]. However, our data showed no significant correlation between the prognosis and the presence of liver cirrhosis in ICC patients. As stratified according to the presence of HBV infection and cirrhosis, the HBV-associated patients achieved relatively prolonged RFS and the presence of cirrhosis revealed no statistically significant prognostic impact. Moreover, a subgroup analysis of cirrhotic patients indicated a tumor size larger than 5 cm to be the foremost factor that significantly reduced the prognosis of cirrhotic patients with ICC.

In the present study, the 1- and 5-year OS rates of 106 patients after hepatic resection were merely at 54.7% and 11.4%, respectively. These survival outcomes might be affected by the patient characteristics, including advanced tumor size and a high proportion of patients with vascular invasion and lymph node metastasis, which were found to be the independent factors that showed significant association with decreased OS. On the other hand, several previous publications demonstrated that HBV infection is associated with survival outcomes of patients with ICC after resection [16–18]. However, due to conflicting results, prognostic impact of HBV infection in ICC remained controversial [19, 20]. In the present study, the recurrence of the tumor was significantly decreased in the HBV carriers (seropositive for HBsAg). In addition, a recent meta-analysis regarding prognostic value of viral hepatitis indicated that HBV infection was associated with relatively low incidence of lymph node metastasis (odd ratio, 0.39; 95% CI, 0.25 to 0.58), which was also found to be an unfavorable prognostic factor for the RFS in this study [21]. Therefore, our data regarding prognostic value of HBV-associated cirrhosis might be affected by inhibiting lymph node metastasis in HBV-associated patients.

To date, information regarding survival outcomes and prognostic factors of cirrhotic ICC remains insufficient due to limited number of studies. A previous relevant publication indicated that hypoalbuminemia, vascular invasion, positive surgical margins, and perioperative blood transfusion are the independent predictors for poor prognosis of cirrhotic ICC [22]. Another study from France indicated that, among 10 patients with unrecognized ICC complicating liver cirrhosis, 5 patients (50%) developed recurrence of the tumor after liver transplantation, suggesting that cirrhosis is associated with more frequent recurrence and poor prognosis in patients with ICC [23]. However, our data revealed that only the tumor size was independently associated with poor prognosis, which was also found to be an independent predictor for the high recurrence rate of the tumor in the entire cohort, rendering us to elucidate that the survival outcomes of the cirrhotic patients with ICC were mainly affected by the recurrence of the tumor. In recent years, preoperative downstaging treatments have been suggested to be performed for patients with HCC in order to meet the Milan criteria or to prevent dropout from the liver transplantation waiting list, as validated by the United Network for Organ Sharing (UNOS) [24]. A previous publication including 35 patients with locally advanced ICC from the University of California, Los Angeles (UCLA), demonstrated that the patients who received both neoadjuvant therapy and adjuvant therapy resulted in relatively better prognosis compared with the patient who received adjuvant therapy only or no therapy (47% versus 33% versus 20%, *P* = 0.03) [25]. More recently, Rayar et al. [26] reported a patient with a huge and locally advanced ICC through preoperative yttrium-90 radioembolization successfully reduced tumor size and then received liver transplantation. Three-year follow-up investigation confirmed that liver function of the patient remained normal and no sign of recurrence was found. Therefore, implementing downstaging treatment for patients with ICC upon hepatic resection might also significantly improve long-term survival of patients with ICC, including patients underlying cirrhosis, which awaits confirmation by future prospective trials.

On the other hand, our results showed no significant difference on prognostic impact between minor hepatectomy and major hepatectomy; the survival outcomes of the patients who received minor hepatectomy revealed to be relatively more favorable without statistical significance. As we performed further subgroup analysis on cirrhotic patients, the impact of major hepatectomy revealed to be a risk factor that significantly reduced both the OS and RFS. However, major hepatectomy was not found to be an independent prognostic factor, which might be on account of the limited number of patients. Hence, we call for future large-scaled trials to verify whether major hepatectomy could be effectively and safely performed in cirrhotic patients with ICC.

Another important factor that significantly affected the survival outcomes was lymph node metastasis, which is a representative hallmark of ICC [27]. Prophylactic lymph node dissection has been considered as a benign factor that might improve the prognosis of patients with ICC [28, 29]. A case report from Japan demonstrated that wide lymph node dissection with hemihepatectomy, including regional and para-aortic lymph nodes, was found to be curative in the first 5-year survival of periductal-infiltrating advanced ICC with para-aortic lymph nodes metastases [30]. In addition, we previously found that there was no significant difference in 5-year survival rates between patients with para-aortic lymph node metastasis and patients with regional nodal metastasis and contended that micrometastases of para-aortic nodes may also play a significant role in the prognosis of patients with biliary cancer [31]. In the present study, the presence of lymph node metastasis showed strong relationship with poor survival outcomes in the entire cohort (*P* < 0.001). In addition, lymph node metastasis also revealed significant difference in the univariate analysis of cirrhotic patients. The survival outcomes of the cirrhotic patients without lymph node metastasis were remarkably higher than the others (1 yr and 5 yr OS: 84.6% and 20.0% versus 41.7% and 0%). Hence, future prospective trials need to focus on the effective and precise detection of lymph node metastasis.

This study had a few underlying limitations. First, this is a retrospective single-center investigation. Limited number of patients might be a weakness. Second, the most common etiological factor for the development of cirrhosis was HBV infection, which is endemic in Asian countries. Therefore, our results need to be validated in large-scaled studies on the basis of other type of cirrhosis, such as primary biliary cirrhosis in western populations.

In conclusion, the presence of cirrhosis in patients with ICC showed no association with the survival outcomes. Tumor size of larger than 5 cm was found to be the foremost factor that significantly influenced long-term survival outcomes. Our results suggest that hepatic resection itself cannot derive favorable prognosis of cirrhotic patients with ICC of larger than 5 cm. Neoadjuvant therapy might improve prognosis of patients with ICC size of larger than 5 cm underlying liver cirrhosis.

## Figures and Tables

**Table 1:** Clinicopathological characteristics of the patients according to the presence of HBV infection and cirrhosis.

Variables	Overall patients (*n* = 106)	Group 1 (*n* = 15)	Group 2 (*n* = 27)	Group 3 (*n* = 10)	Group 4 (*n* = 54)
Age	60.0 ± 10.34	54.9 ± 7.4	54.7 ± 9.1	71.2 ± 8.4	62.0 ± 9.8
Gender					
Male	62 (58.5)	14 (93.3)	14 (51.9)	5 (50.0)	29 (53.7)
Female	44 (41.5)	1 (6.7)	13 (48.1)	5 (50.0)	25 (46.3)
Preoperative AFP					
>9 ng/ml	28 (26.4)	8 (53.3)	9 (33.3)	2 (20.0)	9 (16.7)
≤9 ng/ml	78 (73.6)	7 (46.7)	18 (66.7)	8 (80.0)	45 (83.3)
Preoperative CA19-9					
>35 U/ml	73 (68.9)	9 (60.0)	19 (70.4)	7 (70.0)	38 (70.4)
≤35 U/ml	33 (31.1)	6 (40.0)	8 (29.6)	3 (30.0)	16 (29.6)
Resection type					
Minor hepatectomy	48 (45.3)	10 (66.6)	10 (37.0)	6 (60.0)	22 (40.7)
Major hepatectomy	58 (54.7)	5 (33.4)	17 (63.0)	4 (40.0)	32 (59.3)
Tumor size					
>5 cm	61 (57.5)	7 (46.7)	21 (77.8)	4 (40.0)	29 (53.7)
≤5 cm	45 (42.5)	8 (53.3)	6 (22.2)	6 (60.0)	25 (46.3)
Tumor number					
Single	89 (84.0)	13 (86.7)	23 (85.2)	9 (90.0)	44 (81.5)
Multiple	17 (16.0)	2 (13.3)	4 (14.8)	1 (10.0)	10 (18.5)
Child-Pugh score					
A	100 (94.3)	13 (86.7)	26 (96.3)	9 (90.0)	52 (96.3)
B	6 (5.7)	2 (13.3)	1 (3.7)	1 (10.0)	2 (3.7)
Histologic grade					
Well or moderate	52 (49.1)	5 (33.4)	12 (44.4)	4 (40.0)	31 (57.4)
Poor	54 (50.9)	10 (66.6)	15 (55.6)	6 (60.0)	23 (42.6)
Vascular invasion					
Present	28 (26.4)	8 (53.3)	8 (29.6)	3 (30.0)	9 (16.7)
Absent	78 (73.6)	7 (46.7)	19 (70.4)	7 (70.0)	45 (83.3)
Lymph node metastasis					
Present	48 (45.3)	6 (40.0)	9 (33.3)	6 (60.0)	27 (50.0)
Absent	58 (54.7)	9 (60.0)	18 (66.7)	4 (40.0)	27 (50.0)

Group 1, patients with HBV infection and cirrhosis; Group 2, patients with HBV infection but without cirrhosis; Group 3, patients with cirrhosis but without HBV infection; Group 4, patients without both HBV infection and cirrhosis; AFP, alpha-fetoprotein; CA19-9, carbohydrate antigen 19-9.

**Table 2:** Between-group comparison of the clinicopathological characteristics.

Variables	*P* values					
	I versus II	I versus III	I versus IV	II versus III	II versus IV	III versus IV
Age	NS	<0.001	0.014	<0.001	0.003	0.011
Gender	0.006	0.013	0.005	NS	NS	NS
Preoperative AFP	NS	NS	0.004	NS	NS	NS
Preoperative CA19-9	NS	NS	NS	NS	NS	NS
Resection type	NS	NS	NS	NS	NS	NS
Tumor size	0.04	NS	NS	0.029	0.036	NS
Tumor number	NS	NS	NS	NS	NS	NS
Child-Pugh score	NS	NS	NS	NS	NS	NS
Histologic differentiation	NS	NS	NS	NS	NS	NS
Vascular invasion	NS	NS	0.004	NS	NS	NS
Lymph node metastasis	NS	NS	NS	NS	NS	NS

NS, not significant; AFP, alpha-fetoprotein; CA19-9, carbohydrate antigen 19-9.

**Table 3:** Univariate and multivariate analysis of variables that significantly affected the survival outcomes.

Variables	Overall survival					Relapse-free survival				
	Univariate analysis			Multivariate analysis		Univariate analysis			Multivariate analysis	
	1 yr	5 yr	*P* value	HR (95% CI)	*P* value	1 yr	5 yr	*P* value	HR (95% CI)	*P* value
HBsAg										
Positive	70.0	25.0	0.025	0.665 (0.363–1.220)	0.188	63.3	25.0	0.002	0.505 (0.279–0.914)	0.024
Negative	48.7	6.8				30.3	6.3			
Tumor size										
>5 cm	44.3	5.9	0.002	1.923 (1.147–3.226)	0.013	26.2	5.7	0.003	1.947 (1.177–3.219)	0.009
≤5 cm	68.9	21.4				57.8	19.4			
Child-Pugh score										
A	56.0	12.3	0.005	3.349 (1.387–8.090)	0.007	41.0	11.5	0.017	3.067 (1.293–7.275)	0.011
B	33.3	0				16.7	0			
Vascular invasion										
Present	39.3	4.3	0.049	1.867 (1.103–3.159)	0.020			NS		
Absent	60.3	14.3								
Lymph node metastasis										
Present	37.5	0	<0.001	2.790 (1.628–4.781)	<0.001	18.8	0	<0.001	2.188 (1.310–3.654)	0.003
Absent	69.0	25.0				56.9	22.5			
Tumor number										
Single			NS			43.8	13.2	0.046	1.150 (0.638–2.071)	0.643
Multiple						17.6	0			

HR, hazard ratio; CI, confidence interval; HBsAg, hepatitis B surface antigen; NS, not significant.

**Table 4:** Comparison of the survival curves.

	OS (%)			*P* values				RFS (%)			*P* values			
	1 yr	5 yr	MS	Versus I	Versus II	Versus III	Versus IV	1 yr	5 yr	MS	Versus I	Versus II	Versus III	Versus IV
Group 1	60.0	10.0	12	/	0.540	0.744	0.447	53.3	10.0	12	/	0.718	0.279	0.106
Group 2	66.7	16.7	37	/	/	0.575	0.079	55.6	16.7	24	/	/	0.082	0.011
Group 3	70.0	0	16.5	/	/	/	0.503	30.0	0	6	/	/	/	0.885
Group 4	44.4	11.1	10.5	/	/	/	/	29.6	10.4	3	/	/	/	/

OS: overall survival; MS: median survival month; RFS: relapse-free survival.

**Table 5:** Subgroup analysis of cirrhotic patients (*n* = 25).

Variables	Overall survival					Relapse-free survival				
	Univariate analysis			Multivariate analysis		Univariate analysis			Multivariate analysis	
	1 yr	5 yr	*P* value	HR (95% CI)	*P* value	1 yr	5 yr	*P* value	HR (95% CI)	*P* value
Resection type										
Minor hepatectomy	75.0	14.3	0.004	0.297 (0.051–1.746)	0.179	50.0	11.1	0.019	0.402 (0.093–1.734)	0.222
Major hepatectomy	44.4	0				22.2	0			
Tumor size										
>5 cm	27.3	0	<0.001	16.435 (2.211–122.179)	0.006	9.1	0	<0.001	10.264 (1.881–55.987)	0.007
≤5 cm	92.9	20.0				64.3	14.3			
Lymph node metastasis										
Present	41.7	0	0.002	2.505 (0.595–10.536)	0.210	16.7	0	0.007	2.345 (0.696–7.900)	0.169
Absent	84.6	20.0				61.5	14.3			

HR: hazard ratio, CI: confidence interval.
